# Health-related quality of life for children with rare diagnoses, their parents’ satisfaction with life and the association between the two

**DOI:** 10.1186/1477-7525-11-152

**Published:** 2013-09-08

**Authors:** Heidi Johansen, Brede Dammann, Inger-Lise Andresen, Morten Wang Fagerland

**Affiliations:** 1 TRS National Resource Centre for Rare Disorders, Sunnaas Rehabilitation Hospital, Nesoddtangen 1450, Norway; 2 Unit of Biostatistics and Epidemiology, Oslo University Hospital, PO box 4956, Nydalen, OSLO 0424, Norway

**Keywords:** School children, Disabilities, Rare disorders, Children’s health related quality of life, PedsQL, Parent’s satisfaction of life, SWLS

## Abstract

**Purpose:**

To examine children’s health-related quality of life and parents’ satisfaction with life and explore the association between the two in families where a child has a rare disorder.

**Methods:**

We used a cross-sectional study design. A questionnaire was sent to parents of 439 school children (6–18 years) with congenital rare disorders. Children’s health-related quality of life (HRQOL) was examined by Pediatric Quality of Life Inventory^TM^ 4.0 (PedsQL) Norwegian version. Satisfaction with life was examined by Satisfaction with Life Scale (SWLS).

**Results:**

The response rate was 48% (n = 209). The average age of the children was 12 years and 50% were girls. The parents scored their children with reduced physical, emotional, social and school functioning. The reductions were greatest in the physical area. Parents scored average to high on SWLS but significantly lower than the general Norwegian population. There was a positive association between parental SWLS and the children’s social functioning and school functioning.

**Conclusion:**

Children with congenital, rare disorders often require assistance from many parts of the public service system. Caring for their physical needs should not conflict with their educational and social needs. It is important that the children’s school-life is organized so that the diagnosis does not interfere with the children’s education and social life more than necessary.

## Background

Living with illness and disability can affect everyday life in several ways. Some people with disabilities are continually or periodically in need of specialized medical care. Many need individual facilitation and adaptation in education, work and everyday life. For children, physical disability may lead to reduced interaction with other children because of decreased ability to play and keep up with children without disability, and because of accessibility problems. Cognitive and emotional problems may present challenges in learning and social participation. It has been shown that children with disability have lower health-related quality of life (HRQOL) than most children [1,2] and that many experience challenges in relation to independence and transition into adulthood [3].

Parenting brings both joys and sorrows; however, parents of children with disabilities may face additional strains that affect their health and well-being. A child’s disability can lead to additional work and worries and have an impact on parents’ employment and social life. Parents of chronically ill children may have reduced HRQOL [4,5] and well-being [6]. There is considerable variation in how parents cope with their parental role, and this also applies to parents of children with disabilities [7]. The factors that seem to be related to parents’ HRQOL include degree of emotional support, knowledge about the condition, available leisure time and other family members’ health problems [5].

Research often focused on strains and problems for people living with disabilities, both for the people who are disabled and their family members. This perspective is important to promote understanding for the strenuous aspects of life with a disability and to ensure necessary interventions and services. However, in clinical practice we meet children and adults with disabilities and their family members that live good lives despite the worries and extra strain.

Parental wellbeing can influence their ability to take good care of their children. To our knowledge, no studies have focused on which areas of the disabled children’s lives are most important to their parents’ well-being.

The aims of this study are to examine: (1) HRQOL in school children with seven rare disorders compared with average Norwegian school children; (2) satisfaction with life for parents of children with rare congenital disorders compared with the general population; and (3) the association between parental satisfaction with life and their children’s HRQOL.

## Participants and methods

The study was designed as a cross-sectional postal survey.

### Participants

TRS National Resource Centre for Rare Disorders (TRS) provides services to all people in Norway with some specific rare diagnoses primarily related to the musculoskeletal system. The severity of these disabilities varies, both between the different diagnoses and within the individual diagnoses. Some of the diagnoses are hereditary, none can be cured, but some problems can be prevented and symptoms can be treated. There are seven diagnostic groups: congenital limb deficiency (CLD), arthrogryposis multiplex congenita (AMC), Marfans syndrome (MRF), Ehlers-Danlos syndrome (EDS), short stature due to skeletal dysplasia (StSh), osteogenesis imperfect (OI) and spina bifida/myelomeningocele (MMC).

Persons seeking services at TRS agree to be included in a data-based register. In October 2010 a questionnaire was sent to parents of all children 6–18 years in that register (439 children). After three weeks, a reminder was sent to those who had not responded.

The study was approved by the Regional Committees for Medical and Health Research Ethics in April 2010.

### Methods

The questionnaires were divided into five sections:

1. Parents’ health, education and employment. 2. Children’s diagnosis, medical follow-up, local welfare services and school conditions. 3. Parents’ satisfaction and experience of participation in designing the services. 4. Children’s HRQOL (PedsQL) reported by both the parents and the children. 5. Parents’ satisfaction with life (SWLS).

The Norwegian version of the Pediatric Quality of Life Inventory™ 4.0 (PedsQL) was used [8]. PedsQL is a generic instrument widely used to measure health-related quality of life for children, both reported by the children and by proxy, usually parents [9]. Because the purpose of this study was to explore the association between parents’ satisfaction with life and their perception of their children’s HRQOL, we used the parents’ report. The instrument spans ages 2–18 with age adjusted versions. The 23-item PedsQL can be expressed as a total score or grouped into four domains: physical functioning (eight items), emotional functioning (five items), social functioning (five items), and school functioning (five items). All domains have a scale ranging from 0–100 where higher scores indicate better HRQOL. The PedsQL Norwegian version has shown adequate psychometric properties [8].

We used the Norwegian translation of the Satisfaction with Life Scale (SWLS) to measure the parents’ satisfaction with life [10]. SWLS is an instrument widely used to measure subjective well-being and life satisfaction [11], and it does not focus on problems or strains. SWLS is a self-report questionnaire with five global indicators of quality of life, each scored on a seven-point scale. Results are expressed either as five individual scores (1–7), or a total sum score (5–35) where higher scores indicate better satisfaction with life. We used the sum score in this study. Scores between 20 and 24 are considered an average level of satisfaction with life [12]. The instrument has been tested for reliability and validity and has shown adequate psychometric properties [11].

To compare the PedsQL and SWLS scores from this study with the general Norwegian population, we used PedsQL scores from a study of Norwegian school children [8] and SWLS scores from a Norwegian population-based study [13,14].

### Statistics

We used Glass’s delta effect size (ES) scores to estimate the differences between the scores observed in this study and those of the general Norwegian population. The ES is obtained by dividing the difference in scores by the standard deviation in the general population [15]. Values of ES less than 0.5, from 0.5 to 0.8, and greater than 0.8 are commonly interpreted as indicating small, moderate, and large differences, respectively [16]. One-sample t-tests were used to test the null hypothesis that the ES scores equaled zero. We used paired-samples t-tests to compare PedsQL scores reported by children and parents. Linear regression models were used to examine the influence of different factors on the parents’ SWLS. We considered the following factors: mother’s and father’s education (less than 12 years/12 years or more); mother’s employment (working more than 50% or not); others with health problems in the family (yes/no); child living with both parents (yes/no); the child’s age and gender; and parent-reported PedsQL scores using the four domains of physical, emotional, social and school functioning. P < 0.05 was considered statistically significant.

## Results

The total response rate was 48% and it ranged between 41% and 54% for the different diagnostic groups. There were only small differences in the children’s gender and age among responders and non-responders, both for the total sample and for the individual diagnoses (Additional file 1: Table S1).

Most children (82%) lived with both their mother and father. In 37% of the families another family member had health problems. The mean age of the children was 11.5 years (range 6–18 years). About one third of the children used a wheelchair, and the proportion was highest (83%) among those with MMC (Table 1).

**Table 1:** Socio-demographic and clinical characteristics of the total study sample and the individual diagnostic groups

**Characteristics**	**Total study sample (N = 209)**	**CLD (N = 67)**	**AMC (N = 17)**	**MRF (N = 11)**	**EDS (N = 21)**	**ShSt (N = 28)**	**OI (N = 23)**	**MMC (N = 42)**
**Parents**								
Education level								
Mothers >12 years, number (%)	111 (53)	40 (62)	6 (35)	5 (45)	9 (43)	19 (68)	9 (39)	23 (56)
Fathers >12 years, number (%)	98 (47)	37 (58)	3 (21)	7 (64)	10 (50)	15 (54)	7 (30)	19 (49)
Employment status								
Mother work 50% or more, number (%)	144 (69)	52 (78)	12 (71)	7 (64)	7 (33)	22 (79)	14 (61)	30 (71)
Father work 50% or more, number (%)	184 (88)	63 (94)	13 (76)	8 (73)	18 (90)	25 (89)	19 (83)	38 (91)
Other with health problems in family, number (%)	77 (37)	17 (25)	5 (29)	7 (64)	15 (71)	11 (39)	8 (35)	14 (33)
**Children**								
Girls, number (%)	104 (50)	28 (42)	9 (53)	6 (55)	11 (52)	15 (54)	13 (57)	23 (55)
Age, mean (SD)	12 (34)	11 (35)	12 (34)	14 (32)	13 (32)	10 (35)	12 (32)	11 (32)
School level, number (%)								
Grade 1–6, number (%)	103 (49)	37 (55)	6 (35)	2 (18)	6 (29)	18 (64)	11 (48)	23 (55)
Grade 7–10, number (%)	78 (37)	22 (33)	9 (53)	7 (64)	8 (38)	7 (25)	10 (43)	15 (36)
Grade 11–13 number (%)	27 (13)	8 (12)	2 (12)	2 (18)	7 (33)	3 (11)	2 ( 9)	3 (7)
Living with both parents, number (%)	171 (82)	58 (87)	10 (59)	7 (64)	15 (71)	24 (86)	19 (83)	38 (91)
Manual and/or electric wheel chair, number (%)	80 (38)	7 (10)	10 (59)	1 (9)	3 (14)	15 (54)	9 (39)	35 (83)
Absence from school last year >10 days, number (%)	61 (29)	7 (11)	5 (29)	0	9 (43)	7 (25)	12 (52)	21 (50)

Note: CLD (congenital limb deficiency), AMC (arthrogryposis multiplex congenita), MFS (Marfans syndrome), EDS (Ehlers-Danlos Syndrome), ShSt (short Stature due to skeletal dysplasia), OI (osteogenesis imperfecta), MMC (myelomeningocele).

The total PedsQL score for Norwegian schoolchildren was 86.1, for the total study sample 67.6, varying between 55.9 (MMC) and 78.8 (DSM) for the different diagnostic groups (Additional file 1: Table S2).

On the PedsQL physical, social and school functioning scales, scores were significantly lower than for Norwegian school children, both for the total study sample and for all the diagnostic groups. On the emotional functioning scale the total study sample, EDS, ShSt and MMC groups scored significantly lower than healthy Norwegian children. However, the CLD, AMC, MFS and OI groups had scores comparable with healthy Norwegian children (Table 2).

**Table 2:** PedsQL scores reported by parents compared with PedsQL scores of Norwegian school children (Nsc)

		**Physical functioning**		**Emotional functioning**		**Social functioning**		**School functioning**	
	** N**	**Mean (SD)**	**ES (p-value)**	**Mean (SD)**	**ES (p-value)**	**Mean (SD)**	**ES (p-value)**	**Mean (SD)**	**ES (p-value)**
Nsc	419	88. 8 (11.8)		80.0 (14.1)		88.1 (13.4)		89.0 (12.3)	
Total study sample	209	59.6 (27.2)	2.42 (< 0.001)	71.8 (18,8)	0.58 (< 0.001)	68.8 (22.7)	1.45 (< 0.001)	69.4 (21.1)	1.59 (< 0.001)
CLD	67	77.3 (23.6)	1.00 (< 0.001)	78.5 (17.3)	0.11 (0.477)	80.5 (23.0)	0.57 (0.012)	80.3 (18.6)	0.71 (< 0.001)
AMC	17	47.1 (27.0)	3.53 (< 0.001)	72.7 (20.4)	0.52 (0.186)	61.7 (20.9)	1.97 (< 0.001)	69.7 (23.6)	1.57 (0.007)
MFS	11	73.1 (12.7)	1.33 (0.003)	71.8 (19.1)	0.58 (0.187)	68.6 (13.8)	1.46 (0.001)	66.4 (23.6)	1.84 (0.010)
EDS	21	52.4 (23.3)	3.08 (< 0.001)	59.0 (20.8)	1.49 (< 0.001)	66.0 (23.3)	1.65 (< 0.001)	56.9 (15.3)	2.61 (< 0.001)
ShSt	28	51.2 (21.0)	3.19 (< 0,001)	70.7 (17.1)	0.66 (0,009)	61.9 (18.9)	1.96 (< 0,001)	69.3 (17.7)	1.60 (< 0.001)
OI	23	53.9 (28.6)	2.92 (< 0,001)	73.4 (19.0)	0.47 (0.118)	68.0 (20.7)	1.50 (< 0.001)	71.4 (18.9)	1.43 (< 0.001)
MMC	42	44.5 (24.1)	3.67 (< 0.001)	67.1 (16.7)	0.91 (< 0.001)	59.3 (20.8)	2.20 (< 0.001)	58.4 (21.5)	2.50 (< 0.001)

Note: Some missing values but less than 13% for all variables (Additional file 1: Table S4).

For the total study sample, we observed moderate to large ES scores in all PedsQL domains. Children with CLD had the smallest HRQOL reductions (ES 0.11 to 1.03), while children with MMC had the greatest HRQOL reductions (ES 0.91 to 3.85) compared to Norwegian school children (Table 2).

Both for the total study sample and for the individual diagnostic groups, the children reported better health on nearly all PedsQL scales than did their parents. Most differences were small (moderate on one scale for two groups) (ES 0.00 to 0.67), some were statistically significant (Additional file 1: Table S3).

Parents’ SWLS were significantly lower than the general Norwegian population, both for the total sample (ES = 0.21, p = 0.018) and for the EDS sample (ES = 0.90, p = 0.003), but not for the other diagnostic groups (Table 3).

**Table 3:** SWLS scores of parents compared with SWLS scores of the Norwegian general population (NGP)

	**N**	**Mean (SD)**	**ES (p-value)**
NGP		26.2 (6.3)	
Parents of the total study sample	199	24.9 (6.6)	0.21 (0.018)
CLD-parents	65	26.1 (6.4)	0.02 (0.878)
AMC-parents	15	22.5 (7.1)	0.59 (0.078)
MFS- parents	10	23.6 (5.9)	0.41 (0.228)
EDS-parents	21	20.5 (7.4)	0.90 (0.003)
ShSt-parents	26	25.5 (6.9)	0.11 (0.716)
OI-parents	22	25.7 (5.8)	0.08 (0.855)
MMC-parents	40	25.5 (5.8)	0.08 (0.589)

For the total study population, univariate analyses showed positive associations between parents’ SWLS and all domains of the children’s PedsQL, but no association between parental SWLS and the gender and age of the children. In a multiple linear regression model, we observed significant associations between parental SWLS and others with health problems in the family (p <0.001), children’s social functioning (p = 0.002) and school functioning (p < 0.001) (Table 4).

**Table 4:** Associations between parents SWLS and factors concerning the families and the children

	**Independent variable**	**Univariat regresion**		**Multiple regresion**	
		**Coeffecient (95% CI)**	**p-value**	**Coefficient (95% CI)**	**p-value**
Totale sample (N = 209)	PedsQL Parent report				
	Physical functioning	0.9 (0.6 to 1.1)	< 0.001	−0.4 (−0.8 to 0.06)	0.093
	Emotional functioning	1.6 (1.1 to 2.0)	< 0.001		
	Social functioning	1.2 (0.9 to 1.6)	< 0.001	0.7 (0.2 to 1.3)	0.002
	School functioning	1.6 (1.3 to 2.0)	< 0.001	1.3 (0.8 to 1.8)	< 0.001
	Child gender	4.8 (−13.7 to 23.2)	0.612		
	Child age	−1.5 (−4.2 to 1.3)	0.293		
	Lives with both parents	28.2 (4.8 to 51.6)	0.019		
	Other with health problems in family	−45.9 (−64.1 to −27.7)	< 0.001	−36.5 (−53.2 to −20.0)	< 0001
	Mother work 50% or more	−29.8 (−49.6 to −9.9)	0.003		
	Mother education level >12 years	19.5 (0.8 to 38.3)	0.042		
	Father education level >12 years	29.9 (11.4 to 48.4)	0.002		
CLD (N = 67)	PedsQL Parent report				
	Physical functioning	1.2 (0.6 to 1.9)	< 0.001	−0.4 (−1.5 to 0.7)	0.482
	Emotional functioning	1.6 (0.7 to 2.4)	0.001	−0.3 (−1.5 to 0.8)	0.565
	Social functioning	1.6 (1.0 to2.2)	< 0.001	1.2 (0.04 to 2.4)	0.057
	School functioning	2.0 (1.3 to 2.7)	< 0.001	1.5 (0.3 to 2.7)	0.015
	Child gender	19.7 (−12.3 to 51.7	0.223		
	Child age	2.1 (−2.6 to 6.7)	0.381		
	Lives with both parents	16.9 (−29.4 to 63.2	0.468		
	Other with health problems in family	−20.5 (−57.9 to 16.8)	0.276		
	Mother work 50% or more	−22.0 (−61.0 to 16.9)	0.263		
	Mother education level >12 years	23.6 (−10.2 to 57.4)	0.168		
	Father education level >12 years	29.9 (−3.1 to 63.0)	0.075		
MMC (N = 42)	PedsQL Parent report				
	Physical functioning	0.8 (0.02 to 1.6)	0.044	−0.1 (−1,2 to1,0)	0,906
	Emotional functioning	1.2 (0.1 to 2,3)	0.034	−0.2 (−2.1 to1.7)	0.821
	Social functioning	1.2 (0.3 to 2.1)	0.013	0.3 (−1.5 to 2.0)	0.768
	School functioning	1.4 (0.6 to 2.2 )	0.001	1.4 (−0.1 to 2.9)	0.067
	Child gender	−29.2 (−66.0 to 7.7)	0.117		
	Child age	−1.5 (−7.4 to 4.5)	0.620		
	Lives with both parents	−19.4 (−81.8 to 42.9)	0.532		
	Other with health problems in family	−15.4 (−54.5 to 23.7)	0.431		
	Mother work 50% or more	−8.3 (−49.3 to 32.6)	0.683		
	Mother education level >12 years	−36.4 (−73.2 to 0.3)	0.052		
	Father education level >12 years	40.6 (−36.8 to 44.9)	0.841		

Note: Child gender: girls = 1 and boys =2, lives with both parents: 0 = no and 1 = yes, other with health problems in family: no = 0 and yes = 1, mothers employment level: work 50% or more = 1 and work less than 50% = 2, education level: ≤ 12 years = 1and > 12 years = 2.

For both the CLD and MMC groups separately, we observed significant associations between parental SWLS and all domains of the PedsQL. In multiple regression models, only school functioning remained significant in the CLD group. In the MMC group, none of the domains remained significant, although school functioning had a similar regression coefficient as in the univariate analysis but with a wider confidence interval that barely included zero (Table 4).

## Discussion

This study shows that parent-reported HRQOL is significantly lower for children with rare disorders than for Norwegian school children. The greatest reduction in HRQOL was found in physical functioning, and the smallest reduction was found in emotional functioning. There were considerable differences between the diagnoses.

This study also shows that parents report somewhat reduced SWLS compared to the general Norwegian population. For the total study sample, multiple regression analyses showed significant associations between parental SWLS and the following factors: others with health problems in the family and the child’s social and school functioning. Children’s gender and age did not influence parental SWLS.

Several studies have shown a reduced HRQOL for children with different diagnoses [1,2,17,18]. We expected a similar result for the children in this study. Since they have diagnoses with mainly physical disabilities, we expected the most reduction on the physical scale. This was confirmed for the whole group and for all diagnoses. A physical disability may influence life for a child in many ways: it may lead to some discomfort or pain, give some extra strain and make play with other children difficult. Therefore we expected a reduction also on the emotional scale. This reduction, however, was small, and did not apply to all diagnoses.

Because of the different nature of the diagnoses in this study we expected differences in reported HRQOL, and this was confirmed. The children with MMC, a complex condition with several medical and cognitive challenges [2,19], had lowest scores on all PedsQL scales. On the other hand, the children with CLD, a less complicated diagnosis that influences daily life to a lesser degree than the other diagnoses [20], scored similar to Norwegian school children on most scales.

The fact that a diagnosis is rare can be a burden in itself [21]. However, it is unclear whether this applies to children with rare disorders, or mainly to their parents and adults who themselves have a rare diagnosis. We have not found studies that report PedsQL subscale scores for Norwegian children with common diagnoses, but a study of Norwegian children with CP report total PedsQL scores, parent report [22]. CP is a congenital disorder that gives challenges comparable to those of the diagnoses in this study, especially MMC. The scores for the CP group are similar to those for the diagnoses with lowest scores in this study, MMC and EDS. This may indicate that children with rare disorders do not have lower quality of life than children with well-known common diagnoses. Another interpretation is that PedsQL total score does not capture the specific burdens of having a rare diagnosis.

We expected that having a child with a rare disease would influence parents’ satisfaction with life in a negative way. This was largely confirmed by the fact that the parents in this study had somewhat lower SWLS than the general Norwegian population. However, according to the interpretation of scores [12], the scores observed in this study were between average and high. The mean score for the general Norwegian population is high compared with other countries [13]. That parents of children with disabilities score themselves relatively high on an international scale may reflect that their lives are satisfactory in most ways. However, the reduced score compared with other Norwegians suggests that there are some strains associated with having a disabled child.

There was, however, a difference between the diagnostic groups. For most diagnoses, the parents reported SWLS at nearly the same level as the general Norwegian population. Only parents of children with EDS reported significantly reduced SWLS. This may be explained by the fact that many of the families with EDS had other family members with health problems , which has been shown to give reduced HRQOL [5]. In addition, it can take a long time to establish a diagnosis of EDS, and many feel that they have to struggle to get necessary help from the health- and welfare support system [23].

Many different factors influence satisfaction with life [11,12]. We expected that the better the children function, the more satisfied their parents would be. This was supported by the univariate analyses, where the children’s PedsQL scores on all scales were significantly and positively correlated with parental SWLS.

We also expected that the children’s functioning in different areas of life would influence their parent’s life satisfaction in different degree. In the multiple regression analyses, the most pronounced positive associations were between parental SWLS and the children’s social and school functioning. Our clinical experience is that parents fear that their children may be excluded or bullied because of their diagnoses. Therefore it is logical that the parents’ SWLS is strongly influenced by their children’s social functioning. Parents also worry about how the diagnoses may restrict the child in school [24], because success in school forms the basis for further education and later employment. Education level is strongly associated with participation in the labor force, especially for people with disabilities [25,26]. This may explain why the child’s functioning at school show significant association with parental SWLS.

With increasing age comes an increasing gap in mobility, pace and independence between children with disabilities and their peers [3,27]. This may be of great concern for parents. We expected a negative correlation between the children’s age and parents’ SWLS, but this assumption was not confirmed in this study. Other studies found that parents’ quality of life is reduced in families where more than one family member has health problems [5]. In accordance with this, we found reduced satisfaction with life for parents when other family members had health problems.

### Methodological considerations

There are several methodological and ethical challenges concerning research in rare disorders; it is difficult to find samples of sufficient magnitude [28], and small sample sizes give challenges in relation to anonymity [29].

The study population is recruited through the TRS register. Registration at the center is voluntary with no systematic referral. It is likely that most parents would register their children in order to ensure access to expertise and services at the centre; therefore we assume that most children with these rare disorders are registered at TRS.

The relatively low response rate is usual in self-reported postural cross-sectional studies [30]. There were no significant differences between responders and non- responders in relation to the gender and age of the children. The study samples are small for several diagnoses, and therefore the findings must be interpreted with caution.

Since the aim of this study was to explore associations between parental SWLS and the children’s HRQOL, the parents’ evaluation of their children’s HRQOL was used even though children’s self-reported HRQOL is considered the gold standard [31]. This article does not aim to focus on the problematic side of having a child with disability. Therefore we chose to use a global instrument of life satisfaction to give voice to the coping side of life in families where a child has a rare diagnosis. Not asking both parents to fill out the SWLS separately weakened the study, but it can be justified to use the data as a whole because population studies have found little difference between the sexes [13].

## Conclusion

This study shows that parents of children with rare diagnoses score their children with lower health-related quality of life than the parents of the general population. The greatest reductions in scores are in the physical domain and the lowest reductions in scores are in the emotional domain. Most parents show a similar satisfaction with life as parents of the general population. There were considerable differences between the individual diagnoses for the children’s health-related quality of life and the parents’ satisfaction with life. Associations between children’s school functioning and parents’ satisfaction with life was significant both for the total study sample and for the two diagnostic samples where multivariate analysis was possible.

### Implications

In addition to being an important learning arena, school is also the main arena for participation, social learning and preparation for independent adult life, particularly for children with disabilities. Many need assistance from many parts of the public service system, often simultaneously. Sometimes it seems that the physical needs of these children are taken better care of than their educational and social needs. Health providers may expose children and families for requirements (follow-up, treatment, training) that may conflict with ordinary school life. Prioritizing between the children’s physical, social and educational needs may be difficult. It is important that the children and their parents are involved in decisions about this and about how the children’s school-day is organized.

### Consent

Written informed consent regarding participation in the study was obtained from the parents.

## Competing interests

The authors declare that they have no competing interests.

## Authors’ contributions

HJ, ILA and BD initiated and designed the study and participated in the data collection, the statistical analysis and drafted the manuscript. MWF supervised the study process and contributed to conceptualization and design, data analysis, interpretation of results, drafting and revising the manuscript. All authors participated in the discussions of results in the writing of the paper and read and approved the final manuscript.

## Supplementary Material

Additional file 1: Table S1 Differences between responders and non-responders. **Table S2**: PedsQL total score, parents’ report. **Table S3**: Differences between PedsQL parents’ report and children’s report (observations where both the children’s and parents’ reports are available). **Table S4**: Number of responders in the individual PedsQL scales. Click here for file
