# Supplementary material for: Health-related quality of life for children with rare diagnoses, their parents’ satisfaction with life and the association between the two
Source: Health Qual Life Outcomes. 2013 Sep 8;11:152. doi: 10.1186/1477-7525-11-152 (PMC3844436; doi:10.1186/1477-7525-11-152)
Supplement: Additional file 1: Table S1 — Differences between responders and non-responders. Table S2: PedsQL total score, parents’ report. Table S3: Differences between PedsQL parents’ report and children’s report (observations where both the children’s and parents’ reports are available). Table S4: Number of responders in the individual PedsQL scales. [file 1477-7525-11-152-S1.docx]

**Additional file (data not shown)**

Table S1: Differences between responders and non-responders

|  | Characteristics | Responders | Non- responders |
| --- | --- | --- | --- |
| Total study sample | Number | 209 | 229 |
|  | Gender (girls) number (%) | 104 (50) | 115 (50) |
|  | Mean age (SD) | 11.5 (3.4) | 12.7 (3.2) |
|  |  |  |  |
| CLD | Number (%) | 67 (32) | 87 (38) |
|  | Gender (girls) number (%) | 28 (42) | 46 (53) |
|  | Mean age (SD) | 11.1 (3.5) | 12.6 (3.2) |
|  |  |  |  |
| AMC | Number (%) | 17 (8) | 14 (6) |
|  | Gender (girls) number (%) | 9 (53) | 7 (50) |
|  | Mean age (SD) | 12.2 (3.4) | 11.9 (3.5) |
|  |  |  |  |
| MFS | Number (%) | 11 (5) | 16 (7) |
|  | Gender (girls) number (%) | 6 (55) | 7 (44) |
|  | Mean age (SD) | 13.5 (2.7) | 12.0 (3.8) |
|  |  |  |  |
| EDS | Number (%) | 21 (10) | 28 (12) |
|  | Gender (girls) number (%) | 11(52) | 16 (57) |
|  | Mean age (SD) | 13.3 (3.2) | 13.8 (2.5) |
|  |  |  |  |
| StSh | Number (%) | 28 (13) | 30 (13) |
|  | Gender (girls) number (%) | 15 (54) | 14 (47) |
|  | Mean age (SD) | 10.4(3.5) | 12.1 (3.5) |
|  |  |  |  |
| OI | Number (%) | 23 (11) | 18 (8) |
|  | Gender (girls) number (%) | 13 (57) | 7 (39) |
|  | Mean age (SD) | 11.7 (3.2) | 12.7 (3.4) |
|  |  |  |  |
| MMC | Number (%) | 42 (20) | 36 (16) |
|  | Gender (girls) number (%) | 23 (55) | 17 (47) |
|  | Mean age (SD) | 11.1 (3.2) | 11.8 (4.1) |

Table S2: PedsQL total score, parents’ report.

| Samples | N | PedsQL total score  Mean (SD) |
| --- | --- | --- |
|  |  |  |
| Nsc | 419 | 86.1 (10.2) |
|  |  |  |
| Total study sample | 209 | 67.6 (19.1) |
|  |  |  |
| CLD | 67 | 78.8 (18.5) |
|  |  |  |
| AMC | 17 | 63.6 (14.9) |
|  |  |  |
| MFS | 11 | 70.3 (13.7) |
|  |  |  |
| EDS | 21 | 57.7 (16.4) |
|  |  |  |
| ShSt | 28 | 63.3 (14.4) |
|  |  |  |
| OI | 23 | 66.8 (18.4) |
|  |  |  |
| MMC | 42 | 55.9 (18.2) |

Table S3: Differences between PedsQL parents’ report and children’s report (observations where both the children’s and parents’ reports are available)

|  | **PedsQL domains** | **PedsQL**  **Parent report** | **PedsQL**  **Children’s report** | **Differences** | |
| --- | --- | --- | --- | --- | --- |
| Total study  sample  (N=209) |  | **Mean (SD)** | **Mean(SD)** | **ES** | **(p-value)** |
|  | Physical functioning | 58.2 (26.5) | 62.0 (25.3) | 0.15 | (0.001) |
|  | Emotional functioning | 71.3 (18.4) | 73.7 (20.0) | 0.17 | (0.057) |
|  | Social functioning | 68.4 (22.2) | 75.9 (18.6) | 0.36 | (<0.001) |
|  | School functioning | 68.5 (21.3) | 71.9 (18.0) | 0.14 | (0.005) |
|  |  |  |  |  |  |
| CLD (n=67) | Physical functioning | 74.9(25.5) | 77.4(23.8) | 0.08 | (0.130) |
|  | Emotional functioning | 77.0(17.0) | 78.6(19.6) | 0.12 | (0.482) |
|  | Social functioning | 79.0(23.7) | 82.1(19.6) | 0.13 | (0.197) |
|  | School functioning | 77.4(19.3) | 78.7(16.8) | 0.11 | (0.607) |
|  |  |  |  |  |  |
| AMC (n=17) | Physical functioning | 47.1(22.2) | 47.8(22.8) | 0.05 | (0.740) |
|  | Emotional functioning | 70.7(19.7) | 77.1(17.1) | 0.32 | (0.025) |
|  | Social functioning | 63.6(21.2) | 77.5(14.4) | 0.67 | (0.003) |
|  | School functioning | 69.3(24.4) | 73.6(22.1) | 0.21 | (0.145) |
|  |  |  |  |  |  |
| MFS (n=11) | Physical functioning | 73.4(14.3) | 74.2(16.3) | 0.07 | (0.907) |
|  | Emotional functioning | 73.3(17.0) | 76.7(16.0) | 0.24 | (0.438) |
|  | Social functioning | 71.1(13.6) | 74.4(21.0) | 0.21 | (0.535) |
|  | School functioning | 68.9(24.5) | 71.1(12.2) | 0.08 | (0.805) |
|  |  |  |  |  |  |
| EDS (n=21) | Physical functioning | 54.4(21.0) | 57.7(21.8) | 0.19 | (0.161) |
|  | Emotional functioning | 60.0(22.2) | 65.9(21.4) | 0.27 | (0.218) |
|  | Social functioning | 69.1(20.4) | 79.7(17.9) | 0.55 | (0.007) |
|  | School functioning | 55.3(16.1) | 62.2(16.1) | 0.44 | (0.040) |
|  |  |  |  |  |  |
| StSh (n=28) | Physical functioning | 49.4(19.3) | 57.8(19.5) | 0.47 | (0.004) |
|  | Emotional functioning | 70.2(17.2) | 76.5(20.2) | 0.41 | (0.077) |
|  | Social functioning | 60.7(18.4) | 68.3(20.9) | 0.44 | (0.084) |
|  | School functioning | 69.2(18.1) | 73.5(17.6) | 0.28 | (0.224) |
|  |  |  |  |  |  |
| OI (n=23) | Physical functioning | 49.6(29.1) | 49.7(29.7) | 0.0 | (0.977) |
|  | Emotional functioning | 72.1(18.9) | 68.8(22.9) | -0.17 | (0.455) |
|  | Social functioning | 64.4(21.5) | 72.8(18.5) | 0.41 | (0.005) |
|  | School functioning | 72.0(19.6) | 72.7(16.4) | 0.11 | (0.744) |
|  |  |  |  |  |  |
| MMC (n=42) | Physical functioning | 46.7(24.9) | 52.6(21.9) | 0.24 | (0.042) |
|  | Emotional functioning | 68.5(17.1) | 68.2(18.8) | -0.02 | (0.914) |
|  | Social functioning | 61.8(21.9) | 72.1(13.8) | 0.45 | (0.003) |
|  | School functioning | 58.8(21.5) | 64.3(17.5) | 0.23 | (0.012) |

Note: Some missing values, but less than 29 % for all variables (table d)

Table S4: Number of responders in the individual PedsQL scales

| Instruments | Total study sample  (N=209) | CLD sample  (N=67) | AMC sample  (N=17) | MRF sample  (N=11) | EDS sample  (N=21) | ShSt sample  (N=28) | OI sample  (N=23) | MMC sample  (N=42) |
| --- | --- | --- | --- | --- | --- | --- | --- | --- |
| **PedsQL parent report** | n | n | n | n | n | n | n | n |
| Physical functioning | 200 | 66 | 16 | 10 | 21 | 27 | 20 | 40 |
| Emotional functioning | 201 | 65 | 15 | 11 | 20 | 27 | 22 | 41 |
| Social functioning | 201 | 65 | 15 | 11 | 20 | 27 | 22 | 41 |
| School functioning | 203 | 66 | 15 | 11 | 21 | 27 | 21 | 42 |
| **PedsQL children’s report** |  |  |  |  |  |  |  |  |
| Physical functioning | 167 | 51 | 13 | 9 | 15 | 27 | 18 | 34 |
| Emotional functioning | 170 | 52 | 14 | 9 | 16 | 27 | 18 | 34 |
| Social functioning | 168 | 51 | 14 | 9 | 16 | 27 | 17 | 34 |
| School functioning | 169 | 52 | 14 | 10 | 16 | 27 | 17 | 34 |
